# Exploring physical activity patterns in adolescents with hypermobility spectrum disorder or hypermobile Ehlers-Danlos Syndrome

**DOI:** 10.1186/s12969-025-01124-0

**Published:** 2025-07-08

**Authors:** Elke Schubert-Hjalmarsson, Jonatan Fridolfsson, Daniel Arvidsson, Mats Börjesson, Mari Lundberg

**Affiliations:** 1 Institute of Neuroscience and Physiology, Department of Health and Rehabilitation, Section of Physiotherapy, Sahlgrenska Academy, University of Gothenburg, Gothenburg, Sweden; 2 Department of Physiotherapy, Queen Silvia Children’s Hospital, Sahlgrenska University Hospital, Region Västra Götaland, Gothenburg, Sweden; 3 Center for Health and Performance, Department of Food and Nutrition, and Sport Science, Faculty of Education, University of Gothenburg, Gothenburg, Sweden; 4 Center for Lifestyle Intervention, Department of Molecular and Clinical Medicine, Sahlgrenska Academy, University of Gothenburg, Gothenburg, Sweden; 5 Department of Medicine, Geriatric and Acute Medicine, Sahlgrenska University Hospital, Region Västra Götaland, Gothenburg, Sweden; 6 Pain in Motion (PAIN) Research Group, Department of Physiotherapy, Human Physiology and Anatomy, Faculty of Physical Education & Physiotherapy, Vrije Universiteit Brussel, Brussels, Belgium; 7 Department of Health Promoting Science, Sophiahemmet University, Stockholm, Sweden; 8 University of Gothenburg Centre for Person-Centred Care (GPCC), Sahlgrenska Academy, University of Gothenburg, Gothenburg, Sweden

**Keywords:** Hypermobility, Adolescent, Physical activity, Ehlers-Danlos syndrome

## Abstract

**Background:**

Pain and fatigue are cardinal symptoms in adolescents with Hypermobility Spectrum Disorder (HSD) and Hypermobile Ehlers-Danlos Syndrome (hEDS). Adolescents with HSD/hEDS are assumed to be less physically active as compared to healthy peers, possibly contributing to poorer health, but objectively measured data are lacking. The primary study aim was to investigate physical activity patterns (daytime and nighttime movement behavior) using accelerometers in adolescents with HSD/hEDS versus a control group. The secondary aim was investigation of any association between fatigue and movement behavior, acknowledging pain catastrophizing as a confounder.

**Methods:**

Thirty-seven adolescents with HSD/hEDS and 45 healthy adolescents (aged 13–17 years) participated. Physical activity was measured with Axivity AX3 triaxial accelerometer and an activity-sleep diary was used for assessing time in bed. Fatigue was assessed with the Pediatric Quality of Life Inventory - Multidimensional Fatigue Scale and pain catastrophizing with the Pain Catastrophizing Scale for children.

**Results:**

Adolescents with HSD/hEDS spent significantly more time in sedentary behavior (SED), less time in moderate-to-vigorous physical activity (MVPA) and exhibited significantly more sleep movement during night compared to the control group. An association between fatigue and SED, MVPA daytime or sleep movement in adolescents with HSD/hEDS, with pain catastrophizing as confounder, could not be confirmed.

**Conclusion:**

According to this study, adolescents with HSD/hEDS exhibited physical activity behaviors at levels that are associated to poorer health compared to healthy peers. Measures need to be taken to design health promoting programs for these adolescents, including physical activity and sleep health, using a biopsychosocial approach that considers physical, psychological, and social factors.

**Clinical Trial Registration:**

linicalTrials.gov PRS: Protocol Section NCT05633225.

## Background

Hypermobility Spectrum Disorder (HSD) and Hypermobile Ehlers-Danlos Syndrome (hEDS) are two overlapping heritable connective tissue disorders characterized by joint hypermobility, chronic pain and fatigue. The global prevalence of hEDS is reported at 1:5000 [1], with data for children and adolescents lacking. It is known that adolescents with HSD/hEDS participate less in social activities, report lower health-related quality of life [2, 3], and rate themselves to be less physically active compared to healthy peers [4, 5]. However, in a general paediatric population with asymptomatic general joint hypermobility, self-reported and accelerometry-measured physical activity was not affected [6, 7]. This raises the question of whether fatigue, in addition to the pain, is associated with reduced physical activity in adolescents with HSD/hEDS.

Physical inactivity is a global challenge, with adolescents recommended to be physically active at least 60 min per day at moderate and higher intensity [8]. Moreover, physical activity is a complex behaviour, encompassing sedentary behavior (SED), light intensity physical activity (LIPA), and moderate-to-vigorous physical activity (MVPA) [9]. Both MVPA and SED have been associated with multiple aspects of health, including premature death [10–12].

When measuring physical activity, accuracy and precision are crucial in capturing variations over time [13]. Subjective methods like questionnaires rely on individuals’ estimations, leading to potential measurement uncertainty [14–16]. Objective methods, such as accelerometers, are more reliable, though they are less accurate in capturing certain activities like strength training or swimming [13]. They tend to detect a higher proportion of inactive individuals and show stronger associations with health outcomes [14, 17]. Accelerometers provide detailed data on activity intensity and type, aiding the investigation of healthy activity patterns [13]. To our knowledge, no study has presented movement behavior of adolescents with HSD/hEDS using data from objective methods.

In the general population, there is a clear link between movement behavior and fatigue [18–20], as well as with various indicators of health [21]. Perceived fatigue in adults with different medical conditions is influenced by catastrophizing thoughts [22]. Pain catastrophizing is related to increased pain and disability in both children and adults [23].

The primary aim of the study was to investigate daytime and nighttime movement behaviour using accelerometers in adolescents with HSD/hEDS compared to a control group. The secondary aim was to investigate the association between fatigue and daytime and nighttime movement behaviour with pain catastrophizing as confounder.

## Methods

The study was designed as a prospective, explorative case-control study and is part of a larger study [24], registered at ClinicalTrials.gov PRS: Protocol Section NCT05633225. No separate power analysis was conducted for this exploratory study.

### Participants

Adolescents diagnosed with HSD/hEDS aged 13–17 years were identified through electronic medical records at Children’s Primary Health Care Clinics and two Children Hospitals in western Sweden using the International Classification of Functioning, Disability and Health search terms ‘Q796’ and ‘M357’. Patients’ medical records were preliminarily reviewed to confirm their diagnosis and ensure they met the inclusion criteria.

The participants for the healthy control group were recruited through an advertisement on social media in the region and through a convenience sample via hospital staff. Participants in the control group were matched for gender and age. A one-to-one matching was not conducted due to unequal sample sizes. All participants were included from November 2022 to August 2023.

Participants in both groups were excluded if they were: unable to read or understand Swedish, pregnant, or ≤ 1 year postpartum or neurological/rheumatic/musculoskeletal/metabolic disorders. Potential participants were excluded if they did not meet the 2017 diagnostic criteria for neither HSD nor hEDS [25, 26] or having syndromes like Marfan syndrome, osteogenesis imperfecta, or other EDS sub-types. Control group participants without history of medical health problems or regular use of medications, could not have had pain lasting > 24 h in the preceding three months or engage in elite-level sports.

### Procedures

The adolescents in both groups and their guardians were informed about the study by letter and invited to participate. In this letter, control-group participants were informed of the requirement to be healthy and pain-free. All participants and their guardians were offered additional information orally. Participants under 15 years of age gave verbal assent, while participants over 15 years of age and all guardians provided written informed consent before being invited to the clinic. The individual’s assessment was conducted on a single occasion.

Demographic measures, including height, weight, and the presence of pain on the day of examination were assessed. Participants underwent a medical examination by an independent physician, blinded to group affiliation, following the 2017 criteria for HSD/hEDS which include the Beighton scale for measuring joint hypermobility [26, 27]. Next, they received an accelerometer with instructions, and thereafter, they completed the questionnaires.

### Outcome measures

**Physical activity** was measured using the Axivity AX3 triaxial accelerometer (Axivity Ltd, UK), accompanied by a diary to record times engaged in planned activities, removal of accelerometer, and bedtimes and wake-up times. Data were collected at a rate of 50 Hz and an acceleration range of ± 8G (1G = 9.81 m/s²). The accelerometer measures 23 × 32.5 × 7.6 mm, weigh 11 g, and is water-resistant up to 1.5 m for 1 h. The AX3/AX6 OMGUI Configuration and Analysis Tool was used for programming, downloading, and visualizing data. The accelerometer was attached to the right thigh, 10 cm below the groin on the medial front thigh, using medical tape, for seven days. To minimize the risk of skin reactions, the accelerometer edges were rounded and padded, and tape for sensitive skin was used. In a standing position, the accelerometer was oriented with the x-axis vertical, the y-axis horizontal in the medial-lateral direction, and the z-axis horizontal in the anterior-posterior direction. Raw accelerometer data was processed to measure movement intensity (milligravity, mg) in 3-second epochs using the Frequency Extended Method, categorizing activity into sedentary (SED), light physical activity (LIPA), moderate-to-vigorous physical activity (MVPA) [28]. All data were separated into daytime and nighttime, with nighttime defined as self-reported time in bed. A specific variable representing movement during sleep was created and termed sleep movement, defined as time spent in the SED intensity range during self-reported sleep excluding time with zero acceleration. To visualize differences in physical activity patterns between groups, physical activity intensity was divided into an intensity spectrum [29].

### Patient reported outcome measures (PROMS)

Fatigue was assessed using the Pediatric Quality of Life - Multidimensional Fatigue Scale (PedsQL-F) [30]. The standard version of the child’s self-report for ages 13–18, with a one-month recall period, was used [31, 32]. PedsQL-F comprises 18 questions divided into three subscales: General Fatigue, Sleep/Rest Fatigue, and Cognitive Fatigue, each with six questions, addressing different aspects of fatigue: The General Fatigue domain includes questions about feeling tired, being too tired to spend time with friends, and having difficulty starting or completing tasks.

The Sleep/Rest Fatigue domain covers issues such as sleeping a lot, having trouble sleeping through the night, needing to rest, and spending a lot of time in bed. The Cognitive Fatigue domain includes questions about difficulties with attention, remembering things, and thinking quickly. Responses range from 0 (never a problem) to 4 (almost always a problem), converted to a 0–100 scale. Higher scores indicate less fatigue. The scale demonstrates good reliability and validity in pediatric rheumatology [32].

The Pain Catastrophizing Scale for children (PCS-C) assesses catastrophic thoughts in children and adolescents with pain [33]. It comprises 13 statements beginning with “When I have pain.“. The scale includes three subscales: rumination (4 statements), magnification (3 statements), and helplessness (6 statements). Responses range from 0 (not at all) to 4 (extremely), with scores ranging from 0 to 52. Higher scores indicate greater pain catastrophizing. The PCS-C demonstrates good to excellent internal consistency and predictive as well as concurrent validity in pediatric populations [33, 34].

### Socio-demographic and background information

Socio-demographic information and relevant background variables were gathered using a self-developed questionnaire. The questions asked covered areas such as pain duration and pain frequency.

### Statistical analysis

Control for normal distribution was carried out for descriptive data, physical activity intensity category and questionnaires with the Shapiro-Wilk test and PP-Plot. Data on the ordinal-, interval- and quote level are reported as median and interquartile range. Descriptive data are reported as median and interquartile range or number and percent (%). For the primary aim, a general linear model, with physical activity as dependent variable and age, sex, and group affiliation as independent variables, was used to test difference between groups for each physical activity intensity category separately. Daytime activities and sleep movement were analyzed separately. Normality of residuals are presented in a PP-Plot. Skewed data were log-transformed before entry into the model.

For the secondary aim, two general linear models were used. The first general linear model has physical activity as a dependent variable with age, sex, PCS-C, fatigue, and group affiliation as independent variables, testing for dependence between fatigue and each physical activity intensity category.

The second model has fatigue as dependent variable with age, sex, PCS-C, sleep movement, and group affiliation as independent variables, testing for any dependence between sleep movements and fatigue.

The same models as used for the secondary aim were applied for the analysis within the HSD/hEDS group, but without group affiliation as an independent variable.

For the secondary aim models, results are presented with p-values calculated using robust standard errors to adjust for the correlation between fatigue and PCS-C.

The between-group analysis for ordinal data was performed with the Mann-Whitney-Test (MWU). Estimates of effect size is provided in Cohen´s d (d) for MWU statistic and partial η^2^ and R^2^ for the different general linear models. To interpret the effect sizes: for Cohen’s d, values of 0.2, 0.5, and 0.8 indicate small, medium, and large effects, respectively; for η², thresholds of 0.01, 0.06, and 0.14 indicate small, medium, and large effects, respectively [35]. Statistical analysis was performed with IBM SPSS statistic 28 (Statistical Package for the Social Sciences) (IBM SPSS Data Collection).

## Results

### Socio-demographic and background information - Study cohort

**Fig. 1:**
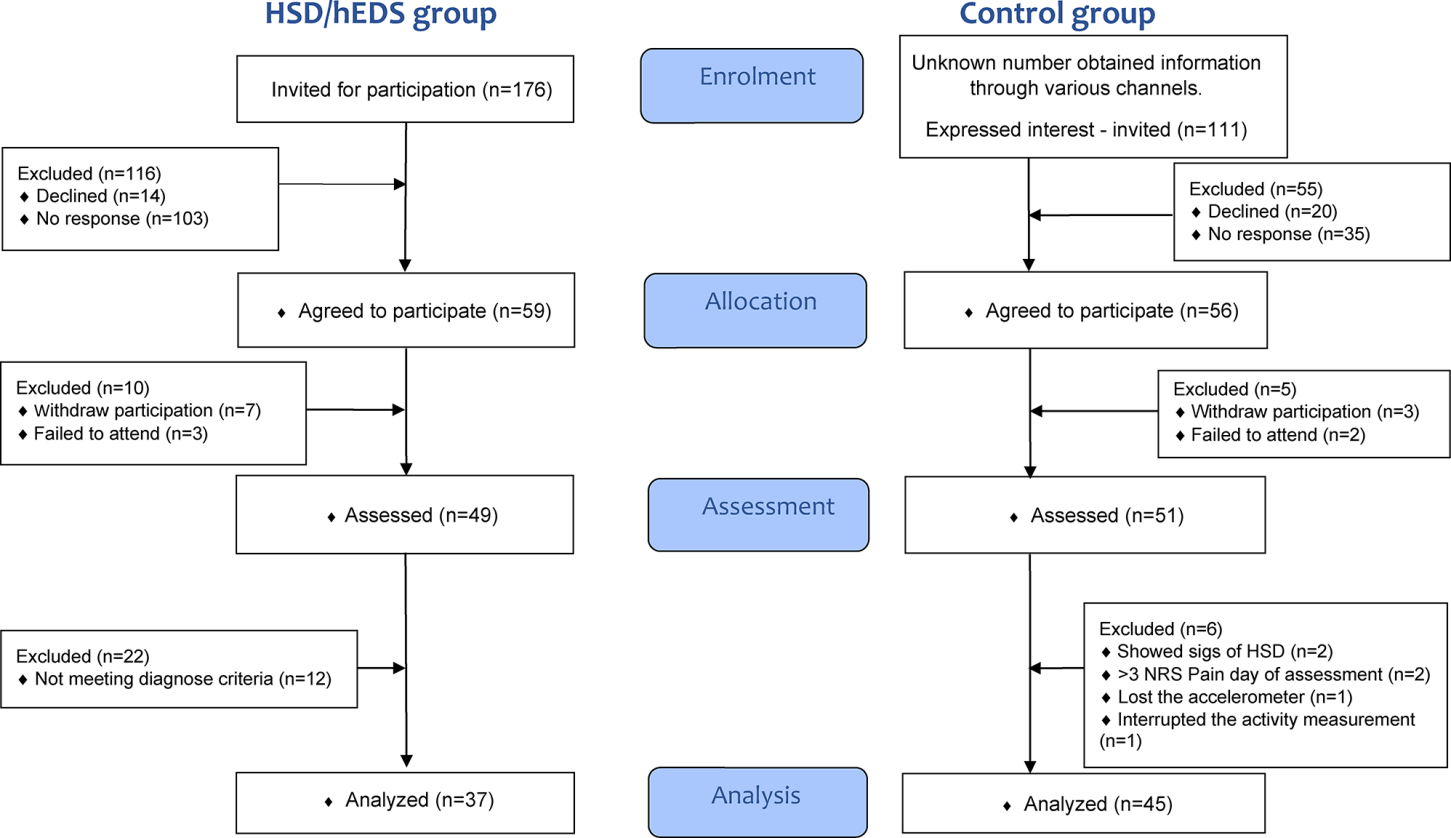
Flow diagram of participants

A total of 176 adolescents with HSD/hEDS and 111 healthy adolescents for the control group were invited to participate in the primary study. Of these, 49 adolescents with HSD/hEDS and 51 healthy adolescents participated. Twelve patients and six control participants were excluded (Fig. 1). A total of 37 adolescents with HSD/hEDS and 45 healthy adolescents participated, which accumulate a total of 323 24-hour periods in the HSD/EDS group and 402 24-hour periods in the control group.

There was no significant difference between the groups in terms of gender distribution (*p* = 0.791) or BMI (*p* = 0.244). The adolescents with HSD/hEDS where older in age (*p* = 0.028) and, as expected, had higher points in the Beighton score (*p* < 0.001) than the control group (Table 1).

**Table 1:** Description of the study population

		Patient group (*n* = 37)	Control group (*n* = 45)	Test statistic ^a^	*P* value ^a^
		Number (%)	Number (%)		
**Gender**	male	10 (27)	11 (24)	811.0	0.80
	female	27 (73)	34 (76)		
		Median (IQR)	Median (IQR)		
**Age (years)**		16.4 (14.8–17.1)	15.4 (14.1–16.3)	597.0	0.03*
**Beighton score (0–9)**		6 (4–6)	2 (1–4)	314.0	< 0.001*
Beighton score male		5 (2–6) ^*n*=10^	1 (0–3) ^*n*=11^	18.5	0.01*
Beighton score female		6 (4–7) ^*n*=27^	2 (2–4) ^*n*=34^	164.5	< 0.001*
**Length (m)**		167.0 (160.8-172.5)	169.0 (164.0-174.4)	694.5	0.20
**Weight (kg)**		58.5 (50.8–72.9)	57.8 (51.9–68.2)	807.0	0.81
**BMI (kg/m²)**		21.2 (18.5–25.6)	20.1 (18.9–23.4)	707.5	0.24

*Abbreviations* BMI = Body Mass Index; IQR = inter quartile range

*Note* ^a^ Mann Whitney U test statistic for group differences; *Statistically significant at α = 0.05

Pain was significantly more common over the past week (*p* < 0.001, MWU = 112, d = 2,21) in the HSD/hEDS group. Thirty-two (71%) adolescents in the control group indicated no pain duration, 2 (4%) less than 3 months, 3 (7%) 3–6 months and 8 (18%) more than 6 months. All adolescents with HSD/hEDS indicated a pain duration more than 3 months. One (3%) 3–6 months and 36 (97%) more than 6 months.

Most adolescents in the control group reported no pain (78%) or only local pain (22%), while most adolescents with HSD/hEDS indicated pain in at least two body quadrants (22%) or widespread pain (57%).

There was a significant difference between the groups, with the HSD/hEDS group perceiving more fatigue (lower PedsQL-F score) (*p* < 0.001) and more pain catastrophizing (higher PCS-C score) (*p* < 0.001) (Table 2).

**Table 2:** Descriptive data of the PROMS, measuring fatigue and catastrophizing

	Patient group (*n* = 37) Median (IQR)	Control group (*n* = 45) Median (IQR)	Test statistic ^a^	Estimate of effect size ^b^	*P* value ^a^
PedsQL-F total score	125 (90–161)	217 (167–240)	216.0	1.64	< 0.001*
PedsQL-F general fatigue	33 (21–46)	75 (63–92)	130.5	2.09	< 0.001*
PedsQL-F sleep/rest fatigue	46 (31–56)	63 (44–75)	448.0	0.86	< 0.001*
PedsQL-F cognitive fatigue	46 (33–58)	75 (58–88)	350.0	1.14	< 0.001*
PCS-C total score	17 (12–29)	4 (0.5–10)	205.0	1.69	< 0.001*
PCS-C rumination	7 (4–11)	2 (0–5)	294.0	1.33	< 0.001*
PCS-C magnification	3 (1–5)	0 (0–2)	388.0	1.03	< 0.001*
PCS-C helplessness	8 (5–11)	1 (0–3)	177.0	1.83	< 0.001*

*Abbreviations* PedsQL-F = Pediatric Quality of Life Inventory- Multidimensional Fatigue Scale child version; PCS-C = Pain Catastrophizing Scale for Children

*Note* ^a^ Mann Whitney U test statistic for group differences; ^b^ Cohen´s D for effect size; *Statistically significant at α = 0.05

### Movement behavior

**Fig. 2:**
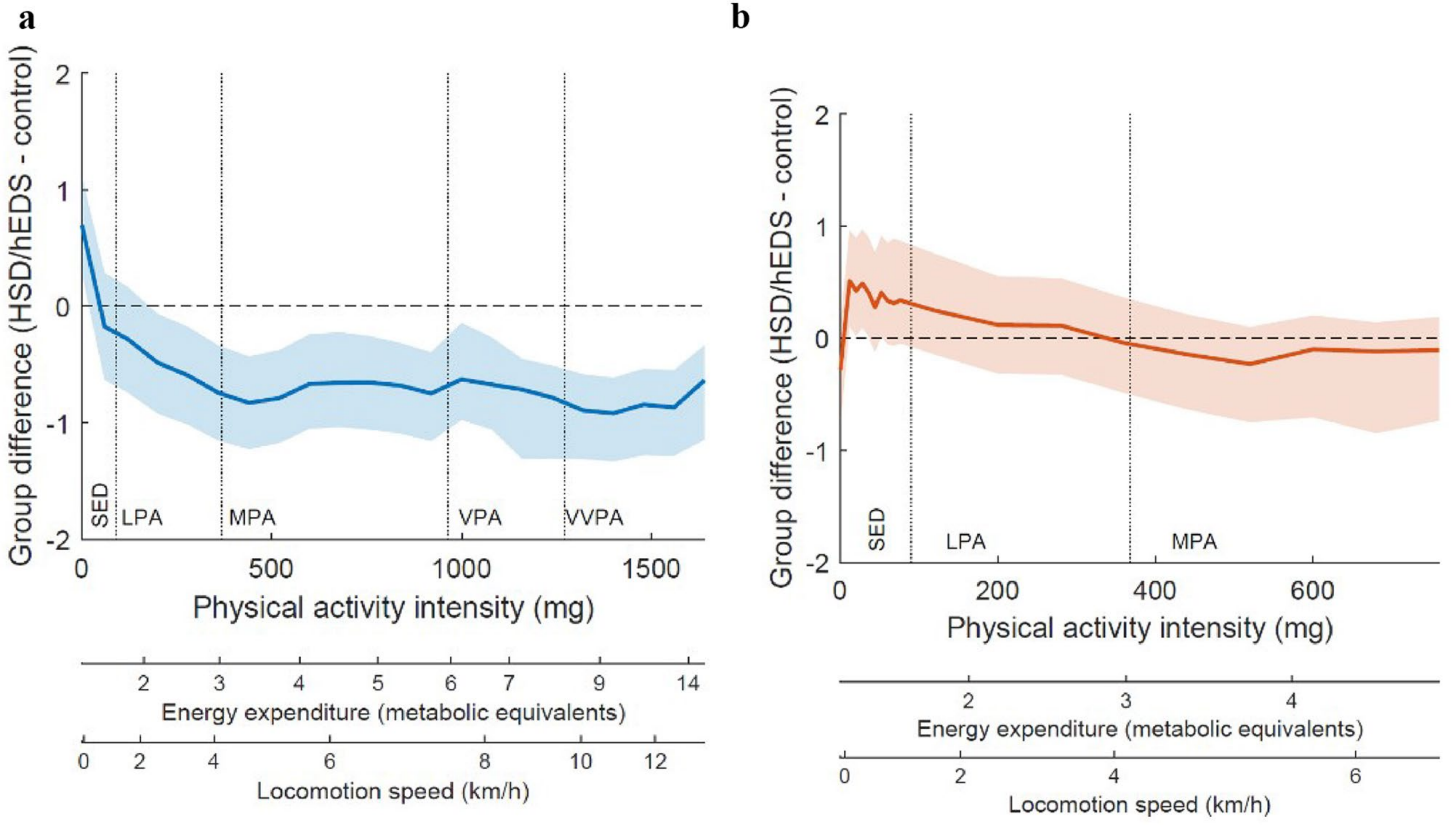
(**a**) Daytime physical activity pattern. (**b**): Nighttime physical activity pattern. *Note*: Figure 2a and b display the disparity in activity levels, separating daytime and nighttime. Positive values on the Y-axis indicate higher activity levels in the patient group, while negative values suggest higher activity levels in the control group. The solid line represents the median difference between the two groups, while the light blue and light orange areas represent the respective confidence interval (95%). Values are expressed in standard deviations

During daytime the HSD/hEDS group spent significantly more time in SED (*p* = 0.010) and less time in MVPA (p = < 0.001) with a median daily MVPA time of 20 min in the HSD/hEDS group and 32 min in the control group (Table 3; Fig. 2a).

During nighttime, the HSD/hEDS group had significantly more sleep movements than the control group (*p* = 0.034) (Table 3; Fig. 2b).

**Table 3:** Time spent at different intensity levels

Category	Patient group (*n* = 37) Median (IQR)	Control group (*n* = 45) Median (IQR)	Partial η^2a^	*R* ^2a^	*P*-value
Sleep (min/day)	914.5 (854.5–957.0)	916,7 (896.7- 969.4)	0.081	0.15	0.01*
Sedentary behavior (min/day)	457.9 (457.9-502.2)	414.0 (382.6- 452.7)	0.146	0.15	< 0 001*
Light intensity physical activity (min/day)	55.1 (39.6–70.9)	65.5 (49.0-72.5)	0.007	0.15	0.46
Moderate-to vigorous physical activity (min/day)	19.7 (19.7–26.0)	31.9 (24.2–44.3)	0.179	0.30	< 0.001*
Sleep movement (Precent av nighttime)	3.4 (2.8–4.9)	3.0 (2.2–3.7)	0.056♦	0.13♦	0.03*♦

*Abbreviations* IQR = inter quartile range

*Note* ^a^ General linear model statistics, data adjusted for age, sex; ♦ based on log-data adjusted for age, and sex; *Statistically significant at α = 0.05

### Dependence between movement behavior and fatigue

There was no statistically significant association between perceived fatigue and daily SED or MVPA in the HSD/hEDS group (*p* = 0.435, β = 0.118; *p* = 0.128, β= -0.691) or in all participants (*p* = 0.717, β = 0.036; *p* = 0.779, β = 0.1). There was no statistically significant association between sleep movements and perceived fatigue in the HSD/hEDS group (*p* = 0.496, β=0.009) or in all participants (*p* = 0.337, β = 0.006).

 There was a statistically significant association between perceived fatigue and PCS-C for the HSD/hEDS group (*p* < 0.001, b= -2.822, partial η^2^ = 0.407) and for all participants (*p* < 0.001, b= -2.720, partial η^2^ = 0.259).

## Discussion

The primary aim of this study was to investigate daytime and nighttime movement behavior using accelerometers in adolescents with HSD/hEDS compared to a control group. The study demonstrated that adolescents with HSD/hEDS spend significantly more time in SED and less time in MVPA at daytime as compared to healthy peers. Additionally, significant differences were found in nighttime movement behavior, finding adolescents with HSD/hEDS moved significantly more during the night. The secondary aim was to investigate the association between fatigue, with pain catastrophizing as a confounder, and daytime or nighttime movement behaviour. Our study could not show an association between perceived fatigue and daytime or nighttime movement behaviour.

### Movement behavior - daytime

 In our study, neither group reached the recommended health-promoting levels of physical activity for adolescents. Furthermore, adolescents with HSD/hEDS spent significantly more time in SED and less time in MVPA compared to the control group. Therefore, they specifically did not meet the recommended levels of physical activity for adolescents, which is associated with an increased risk of developing non-communicable diseases such as cancer, cardiac conditions, and diabetes [8]. Pain and fatigue have been put forward as a barrier for low physical activity levels as well as psychological factors [36]. Therefore, it is important to develop sustainable interventions that facilitate more physical activity that maintaining activity levels over time.

 Few studies have examined the effect of physiotherapy interventions in adolescents with HSD/hEDS. In two randomized controlled trials with exercise programs targeting strength and stability in 7–16-year-olds with HSD/hEDS, both studies interventions had a positive effect on pain and function after six weeks respectively 16 weeks [37, 38]. However, to our knowledge no study has investigated long-term interventions aimed at increasing physical activity in adolescents with HSD/hEDS. Telford et al. (2023) debate the importance av LIPA as a replacement for SED as a first step to increase daily movement and to facilitate physical activity at higher intensities in adolescents in general [39]. Physiotherapy interventions generally have positive effects on pain [37, 38], which can facilitate participation in everyday activities such as walking and cycling, thereby promoting physical activity in adolescents with HSD/hEDS. Further randomized controlled trials in adolescents incorporating long-term (> 12 month) follow-up are needed.

### Fatigue and movement behavior - daytime

 Our study could not show an association between perceived fatigue and higher levels of SED and lower levels of MVPA at daytime. This may be due to the exploratory nature of this study with the potential risk of being underpowered. However, we could show a statistically significant correlation between fatigue and pain catastrophizing. Pain and fatigue have been put forward as a barrier for exercise in adults with HSD/hEDS, alongside with psychological factors [36]. A four-year-long interdisciplinary pain treatment program for adolescents with disabling chronic pain has shown a long-term effect in 60% of adolescents on pain-related and psychological outcome measures, including pain catastrophizing [40]. There is currently limited evidence for physical therapy interventions in adolescents with HSD/hEDS [41], highlighting the importance of research on long-term treatment plans. These interventions should include multiple psychological strategies to improve physical activity levels in adolescents with HSD/hEDS.

### Fatigue and movement behavior - nighttime

 In our study, we could not discern whether adolescents with HSD/hEDS, who have more sleep movements, reported higher levels of perceived fatigue. However, there could be other causes beyond sleep movements that may affect perceived fatigue. We did not further investigate if there is an association between sleep movements and pain severity or psychological factors like anxiety or depression. In an American cohort the prevalence of prolonged fatigue was 3%, with 1.6% prolonged fatigue with a depressive or anxiety disorder [42]. This leads to the question of whether psychological factors are those that can affect both sleep movements through a motor restlessness and the perceived fatigue reported by adolescents with HSD/hEDS.

 Palermo et al. (2008) showed that lower sleep efficiency in adolescents was a significant predictor of greater activity limitations [43]. We did not control for sleep disorders, but sleep disorders could be one explanation for the measured sleep movements in our study [44]. Interrupted or poor sleep has also been shown to be associated with developing chronic pain and can be an obstacle to successful pain treatment in children and adolescents [45, 46]. Therefore, it is important to examine sleep, sleep health, and sleep difficulties more thoroughly in adolescents with HSD/hEDS.

### Strength and limitations

 To our knowledge, this is the first study investigating physical activity in adolescents with HSD/hEDS using objective methods such as accelerometers, which provide more reliable data on the intensity of physical activity and activity patterns. Because of the exploratory nature of this study, focusing on movement behavior, we did not collect data on sleep quality, sleep difficulties, or cognitive/psychological factors like anxiety, depression, and pain severity. These data would be valuable in a more comprehensive study design.

 Another limitation is that the data used in this study are derived from a larger study, and there was no separate power analysis specifically for this part of the study. We found significant differences in physical activity levels but with a low to moderate effect size (partial η²), which means the results should be interpreted with caution. This may also explain why we could not establish any relationship between fatigue and SED or MVPA or between sleep movements and fatigue in this study.

## Conclusion

 According to this study, adolescents with HSD/hEDS exhibited physical activity behaviors at levels that are associated to poorer health compared to healthy peers. An association between fatigue and daytime movement or sleep movement in adolescents with HSD/hEDS could not be confirmed. Medical and psychosocial health issues—such as chronic pain, anxiety, and sleep disturbances—may play a role in these patterns and should be taken into account when interpreting the results. More research is necessary to gain a deeper understanding of the significance of these results, if any. Measures need be taken to design health promotion programs for these adolescents that include increased physical activity and improved sleep health, using a biopsychosocial approach that considers physical, psychological, and social factors.

## Data Availability

The datasets used and/or analysed during the current study are available from the corresponding author, Elke Schubert-Hjalmarsson, on reasonable request.

## Abbreviations

HSD: Hypermobility Spectrum Disorder
hEDS: Hypermobile Ehlers-Danlos Syndrome
SED: Sedentary behavior
LIPA: Light intensity physical activity
MVPA: Moderate-to-vigorous physical activity
PedsQL-F: Pediatric Quality of Life Multidimensional Fatigue Scale child version
PCS-C: Pain Catastrophizing Scale for Children
PROM: Patient-reported outcome measures
